# Linkages between service recovery system and customer justice perceptions: A multi-level model of employee service recovery performance

**DOI:** 10.12688/f1000research.135103.1

**Published:** 2023-08-07

**Authors:** Abhishek S Rao, Yogesh Pai P, Lakshminarayanan Sethumadhavan

**Affiliations:** 1 Department of Commerce, Manipal Academy of Higher Education, Manipal, Karnataka, 576104, India; 2 Department of Humanities and Management, Manipal Institute of Technology (MIT), Manipal Academy of Higher Education, Manipal, Karnataka, 576104, India; 3 Manipal Institute of Management, Manipal Academy of Higher Education, Manipal, Karnataka, 576104, India

**Keywords:** Service Recovery System, Job Resources, Job Demands, Burnout, Work Engagement, Customer Justice Perception

## Abstract

Past research aimed at service recovery has focused on actions that are taken to retain customers, and the possibility of overcoming the mistakes of service delivery, though the multidimensional nature of the service recovery process has begun to move beyond the complaint handling process. In this paper, we identify the most important issues addressed in service recovery and present a framework for addressing them

We used an extensive integrative review methodology. Between 1988 and 2017 the number of articles in these journals was kept between 26 and 30. The majority took a business perspective to study services recovery, while a minority took an inter-disciplinarity perspective. This research aims to investigate the relationship between the constructs in the JD-R Model and employee service recovery performance, and to adopt a multi-actor research design for more accurate measurement. The findings of this study are expected to provide valuable insights into the antecedents and consequences of service recovery performance in the context of the service recovery system

This research contributes to the discourse on service recovery on several fronts. First, this research answers the call for building a multi-level model of relevant constructs to explain the service recovery performance of frontline employees in the hospitality sector. Thus, this research is expected to contribute to robust conclusions on the antecedents and consequences of employee service recovery performance nested within the unit-level construct, i.e., the service recovery system.

## Introduction

Service recovery constitutes ‘the actions taken by a service provider aimed at resolving failures’ ( Smith, Karvan, & Markland, 2009). Retaining current customers who demonstrate dissatisfaction and the possibility of overcoming mistakes in service delivery are pointed out as the benefits of service recovery. Research on service recovery is partly grounded in the marketing literature that has examined the impact of service recovery practices on customer satisfaction. However, the multidimensional nature of the service recovery process has begun to go beyond the complaint-handling process and to study the structural dimensions of the service recovery system ( Davidow, 2003) while distinguishing the structural dimensions from the mere ‘infrastructural dimension’ of the service recovery process. Consistent with this trend of the interdisciplinary approach adopted in the research discourse on service recovery, this research proposal brings together the variables in service operations and organizational behavior to empirically test a theorized multi-level model that aims to bridge the macro and micro perspectives of service recovery.

### The crucial role of frontline employees

In this connection, Ryan & Ployhart (2003, p. 377) have argued that “a focus on the customer has become a major component of organizational strategies, regardless of whether the organization is in the service or manufacturing sector.” Therefore, frontline employees’ performance ensuring customer satisfaction and customer justice perceptions is crucial for restoring dissatisfied customers to a pre-service failure state. Accordingly, Liao (2007) argued that “frontline employees, placed at the organization-customer interface and directly responsible for the production and delivery of service, act as boundary spanners for the service company” ( Bettencourt & Brown, 2003). Therefore, it is necessary to study the interaction between employees’ service recovery performance and resultant customer justice perceptions.

### Importance of customer justice perceptions

One of the goals of service firms is to build customer loyalty ( Heskett *et al.*, 1994). Against this backdrop, it is essential to note that the experience of service failures in the service process leads to customer defects ( Hart *et al.*, 1990). Prior research has argued that customer defections are influenced by adverse customer justice perceptions concerning firms’ service recovery processes. It is essential to study the customer justice perceptions as this construct is linked to several other variables such as relationship quality ( Aurier & Siadou-Martin, 2007), repatronage intension ( MartÃ­nez-Tur *et al.*, 2001; Teo Thompson & Lim Vivien, 2001; Maxham & Netemeyer, 2002; Davidow, 2003; Witrz & Mattila, 2004; Kim, Kim & Kim, 2009; de Matos, Vieira & Veiga, 2012; Ro and Olson, 2014; Xu *et al.*, 2014; Kim & Tang, 2016), satisfaction ( Murray, 2006; del Río-Lanza, Vázquez-Casielles & Díaz-Martín, 2009; Nikbin & Hyun, 2014; Ro & Olson, 2014; Waqas, Ali & Khan, 2014; Paper *et al.*, 2016), overall satisfaction ( Vázquez-Casielles *et al.*, 2010), loyalty ( Kim & Kandampully, no date; Chebat and Slusarczyk, 2005; DeWitt, Nguyen & Marshall, 2008; Abbas, Abdullateef & Mokhtar, 2015), exit ( Chebat & Slusarczyk, 2005), negative word of mouth ( Teo Thompson & Lim Vivien, 2001; Ro & Olson, 2014), positive word of mouth ( Kim, Kim & Kim, 2009; Choi & Choi, 2013), word of mouth ( Maxham & Netemeyer, 2002; Witrz & Mattila, 2004), continuous usage ( Zhou, 2015), switching intension ( Nikbin *et al.*, 2012), and trust ( Kim, Kim & Kim, 2009). Interestingly, these customer justice perceptions are linked to an organization’s future business performance.

### Need for a standardized service recovery system

This overwhelming evidence that indicates the organizational importance of customer justice perceptions in the service recovery context has led to the increasing need to formalize the service recovery system that guides and trains frontline employees in their service recovery performance and places the systems and processes in this regard. Formalizing and institutionalizing the service recovery system assumes importance in the context of its role in determining customer justice perceptions influenced by employee service recovery performance. Therefore, this research proposal intends to examine the impact of the service recovery system, conceptualized and operationalized at the organizational level, on the perceptions of customer justice that arise in the service recovery process.

### Why it is essential to focus on the behavior of frontline employees

In this context, hospitality sector firms have begun to invest heavily in modifying frontline employees’ behavior ( Stock *et al.*, 2017). In this connection, the importance of the role of frontline employees arises due to the dyadic roles that they play with customers and the social exchange processes that they initiate, which determine the customer evaluations of employee performance, in particular, the company performance in general ( Solomon *et al.*, 1985), and the increase in repeat visits and repurchase intentions ( Borucki & Burke, 1999).

### Need for the study: employee service recovery performance and customer justice perceptions

In this connection, the literature on service recovery mentions the service recovery paradox ( Michel *et al.*, 2009) as a potent tool to take customers back to a state of customer delight in such a manner that the satisfaction that they perceive regarding the service provided would be even higher than the satisfaction that they were experiencing prior to service failure. Therefore, the extent of efficacy demonstrated by a service organization and its frontline employees in the service recovery process is significant. In this context, frontline employees significantly lead customers to satisfaction, delight, etc. ( Rod & Ashill, 2009). Accordingly, employee recovery performance affects customer justice perceptions, a theoretical relationship that remains empirically unexplored in the hospitality sector.

### Employee service recovery performance and the Job Demands-Resource (JD-R) model

Research on the antecedents of employee recovery performance, which explains the interactive effects of the JD-R Model on employee service recovery performance, is scarce. However, the theoretical premises of employee recovery performance are well articulated, which can be seen in the relationship between burnout and employee recovery performance and between work engagement and employee service recovery performance. For example, scholarly research ( Kahn, 1992; Britt, 1999; Harter *et al.*, 2002; Maslach *et al.*, 1997; Macey & Schneider, 2008) has identified several working conditions as the antecedents of burnout and work engagement. Among these, a few examples of antecedents are job clarity, job control, job relevance, work expectations, supportiveness of supervisors and coworkers, opportunities for growth and development, job demands, rewards and recognition, a community of support, and fairness. The similarity in all these predictors lies in the factors related to working conditions ( Crawford *et al.*, 2010). This similarity is captured in the JD-R Model in the two categories of antecedents: job resources and job demands.

### Why the JD-R model?

The JD-R model extends the demand-control model (DCM) and effort-reward imbalance (ERI) model. Even as the JD-R Model considers the elements of the DCM and ERI Models, it also adds specific elements of job resources and demands. While the DCM and ERI models consider only a few specific factors, such as job demand and job control in the DCM model and effort and reward in the ERI model, the JD-R model considers a whole range of factors that can be encompassed under both job resources and job demands.

### Need for integration of the differentiated job demands perspective to explain service recovery behaviour

Although early research on the relationship between the JD-R Model and the consequences of burnout and work engagement conceptualized only job resources as the antecedent of work engagement and job demands as the antecedent of burnout, later research identified the interaction among job resources, job demands, work engagement, and burnout. However, there were inconsistencies in the empirical findings when this early conceptualization was subjected to empirical testing. Therefore, researchers later found the reasons for these inconsistencies by proposing a differentiated job demand perspective. Accordingly, they argued that job demands consist of ‘challenge stressors’ and ‘hindrance stressors,’ among which challenge stressors increase both burnout and work engagement, whereas ‘hindrance stressors’ increase only burnout. Furthermore, job resources moderated the presence of job demands. Therefore, the higher the level of job resources, the more interactive the effect of job demands on burnout and work engagement. However, this differentiated perspective on job demands has not been integrated into the research discourse examining the burnout and work engagement levels of frontline employees in the hospitality sector, nor has its effect on employee recovery performance.

### Need for multi-level modeling of service recovery in the hospitality sector

Scholarly research on service performance has examined the organization-level factors such as ‘climate’ constructs ( Schneider, 1990) that lead to customer satisfaction or the individual-level factors ( Frei & McDaniel, 1998) to account for individual differences. However, the exclusive concentration at the individual- or unit-level analysis would lead to either missing situational factors ( Kozlowski & Klein, 2000) or not accounting for individual differences ( Liao & Chuang, 2004). There are three essential issues in this context. First, the extent of the influence of factors at a given level after considering the factors at a different level. Second, cross-level interactions among variables of interest are expected to impact endogenous variables. Third, there is a possibility of misspecification, as a result of which spurious relationships are attempted. The impact of a variable at a given level is mistakenly interpreted as exercising its impact on the variable at another level ( Kozlowski & Klein, 2000). Therefore, it is necessary to examine organization-level variables that impact individual-level variables, such as employee recovery performance and customer justice perceptions. Second, it is necessary to understand the cross-level interactions by adopting either a top-down or bottom-up process, the impact of organization-level or group-level variables on individual-level variables, or the impact of individual-level variables on either group-level or organization-level variables. Thus, this understanding of the impact of variables at different levels can only be understood by bridging the macro and micro perspectives ( Kozlowski & Klein, 2000). This study endeavors to conduct an integrated analysis of micro and macro perspectives of the service recovery process in the hospitality sector by investigating the impact of the service recovery system, which is at the organizational level, on employee recovery performance as well as its antecedents and consequences at the individual level of analysis.

### Theory

Investigating the antecedents and effects of employee service recovery performance requires using multilevel theory. A single-level analysis does not capture the interaction between macro- and micro-level constructs. Consequently, the entire gamut of a phenomenon cannot be fully captured. Research discourse on multilevel modeling has consistently argued that a given social phenomenon will likely be nested within higher-level constructs. Therefore, multilevel theory explains the proposed relationships among constructs that may belong to the macro and micro perspectives.

## Methods

We used an extensive integrative review methodology. Integrative reviews comprise research that uses various approaches to achieve various goals, including concept definition, theory review, evidence review, and methodological issue analysis ( Broome, 1993). The goal of the inclusive sampling frame was to provide a thorough understanding of the ideas, theories, and problems significant to service recovery. There were three steps to the complete evaluation procedure. First, we conducted a literature search of the literature for publications published in the Web of Knowledge database using the terms “service recovery,” “complaint handling,” and “complaint management.” In order to find further research on service recovery, we also conducted issue-by-issue searches of the following service journals: Journal of Service Research, Journal of Business Research, Total Quality Management and Business Excellence, Journal of Hospitality and Tourism Research, and many more. Then, using an ancestry technique, we looked for studies’ references that we had previously recognized as being relevant to the present Study. For review, this method produced more than 300 papers. After that, we reviewed the literature. An article was considered appropriate for inclusion if it specifically examined a firm’s response to a customer complaint, excluding articles that only examined customer reactions to service failures or why customers complain.

Additionally, it must have been published in an academic journal undergoing peer review, excluding conference proceedings. First, we assigned each article a tick (if both criteria were met) or a question mark (if we were not sure) or left it unmarked (if neither condition nor both conditions were met). Then, we read every item with a question mark next to it, assessed its relevance and educational value, and checked or unchecked it. Finally, we classified papers under Business Management & Accounting and Marketing as either using an interdisciplinary approach or only depending on one of the two fields looking at service recovery. Between 1988 and 2017, 120 articles from 26 different publications were kept. The majority of papers (75.3%) took a marketing perspective to study service recovery; only a minority took a human resource management (11.1%), operations management (9.4%), or inter-disciplinary perspective (4.2%). The actual literature study was the last step in our process. First, based on the level of analysis (firm, employee, or customer) employed to address service reimbursement difficulties, we developed an overall classification scheme. Then, we grouped the concepts and study findings into the classification scheme. Third, we found connections between ideas related to one level of analysis or several layers of analysis, and we visually demonstrated their logical chain of relationships. Finally, we analyzed the gaps in the literature and identified the research opportunities for future studies. Our comprehensive approach enabled us to gain a deep understanding of the service reimbursement difficulties from various perspectives and identify the key issues that need to be addressed. Moreover, our findings can provide valuable insights for policymakers, resatuarant providers, and researchers to improve the service recovery process and enhance the quality of service for customers. Finally, we conducted a comprehensive literature review to validate and enrich our conceptual map. Overall, our process allowed us to gain a deeper understanding of the complexities of service recovery difficulties and provided a framework for future research in this area.

### Conceptual framework

Accordingly, this Study argues that customer justice perceptions, captured at the customer or individual level of analysis, are influenced by employee service recovery performance. In turn, the construct of employee service recovery performance, captured at the employee or individual level of analysis, is influenced by the impact of job demands and job resources exercised through the mediated effects of burnout and work engagement. Furthermore, the individual-level constructs of job resources and job demands are influenced by the unit-level construct of the service recovery system. This relationship among constructs requires capturing the macro-level construct—the service recovery system’s impact on job resources and job demands—before assessing their impact on employee burnout and work engagement.

Furthermore, the theory of situational strength argues that a given construct, which is initially at the individual level, is likely to exercise its influence on its predictor variables only after its aggregated impact is considered. Prior research on employees’ service recovery performance has led to this advocacy. The implicit reason for advocating the aggregated effect, not the individual effect, of employee service recovery performance is that customers’ service recovery experience will likely result from the service provided by several frontline employees. Therefore, customer evaluation of their experience of the service recovery behavior of frontline employees is the product of the service recovery behavior demonstrated by a given unit’s or restaurant’s frontline employees. Accordingly, the appropriate way to capture employee service recovery performance is to capture it at the unit or restaurant-level instead of at the individual level. In other words, although data on employee service recovery performance is collected at the individual level, it needs to be aggregated at the unit level.

The impact of the service recovery system on job resources and job demands, which integrates macro and micro perspectives, is captured by adopting a top-down process. The construct data relating to employee service recovery performance would be aggregated from the individual to the unit or restaurant level by adopting the bottom-up process. Accordingly, the impact of the restaurant-level construct, i.e., unit-level employee service recovery performance, on customer justice perceptions captured at the individual level will be examined.

**Figure 1.**
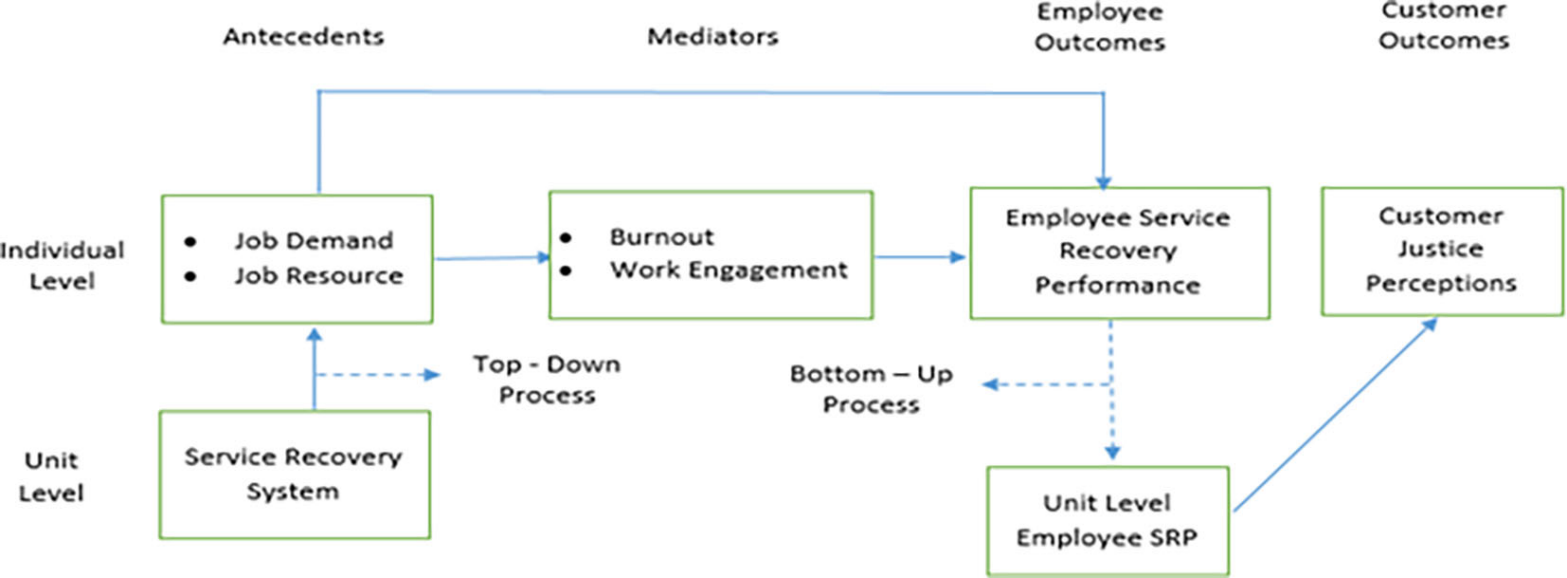
Conceptual framework of the multi-level model of employee service recovery performance.

The following conceptual framework ( Figure 1) presents the proposed relationships of the multi-level model:

### Relationship of the study at the construct level

*Service recovery system*

Many prior studies have used customer data to study aspects of the service recovery process ( Smith, Karwan, & Markland, 2009), even as service providers handle organizations that provide service and service recovery problems. Therefore, the construction of a service recovery system looks at the issue of service recovery from the standpoint of service provider entities.

Focal construct. Therefore, the construct of a service recovery system is defined as the structural dimension of the service recovery system employed by a service organization in its service recovery process. Process formality, decentralization, comprehensiveness, human intensity, system intensity, accessibility, and influence are the seven dimensions of a service recovery system.

**Formality:** Process formality is ‘the degree to which service recovery is controlled by explicit rules, procedures, and norms that dictate recovery activities’ ( Papke-Shields *et al.*, 2002; Pugh *et al.*, 1969).

**Decentralization:** While the concept of centralization is defined as the ‘locus of authority or devolution of responsibilities for handling recovery activities’ in the literature on organizational design and strategy ( Pugh *et al.*, 1969; Segars & Grover, 1998), the concept of decentralization is essentially interpreted in terms of empowerment that gives employees the freedom to overcome mistakes related to the service delivery process ( Michel *et al.*, 2009).

**Comprehensiveness**: The dimension of comprehensiveness is defined as ‘the extent to which attempts are made to be exhaustive or inclusive in considering all potential recovery activities once a failure has occurred’ ( Smith, Karwan, & Markland, 2009), which considers several options for taking an optimal action ( Papke-Shields *et al.*, 2002) and an extensive range of solutions ( McColl-Kennedy & Sparks, 2003).

**Human intensity**: The dimension of human intensity is defined as ‘the magnitude of resources committed to recovery as evidenced by the provision for employee training as well as the extent of employee evaluations’ ( Smith, Karwan, & Markland, 2009; Segars & Grover, 1998; Papke-Shields *et al.*, 2002). This dimension captures the resources needed to train employees in the service recovery process ( Smith, Karwan, & Markland, 2009).

**System intensity**: System intensity is defined as ‘the magnitude of resources committed to tracking and monitoring service failures and recovery efforts.’ ( Smith, Karwan, & Markland, 2009). This is especially helpful in avoiding future mistakes ( Tax *et al.*, 1998).

**Accessibility:** It is defined as ‘the provision for capturing the voice of the customer when failures occur.’ ( Smith, Karwan, & Markland, 2009). This gives customers opportunities for feedback on the quality of service they have received ( Colgate & Norris, 2001).

**Influence:** It is defined as ‘the ability of the system to adapt depending upon the situation and “position” of the customer’ ( Smith, Karwan, & Markland, 2009). This Study examines the service recovery process from the customer’s perspective. Therefore, this dimension captures the degree to which the customer exercises control over the service recovery process and service recovery system ( Tax *et al.*, 1998).

*Employee service recovery performance*

Focal construct. Employee service recovery performance is defined as ‘the behaviors in which customer service employees who directly handle customer complaints engage in the recovery of customer satisfaction and loyalty after service failures’ ( Liao, 2007). Making an apology, problem-solving, demonstrating courteous behavior, providing an explanation, and promptly handling complaints are the five dimensions of this construct.

*Customer justice perceptions*

Focal construct. Customer justice perceptions constitute four dimensions: distributive, procedural, informational, and interpersonal. Distributive justice refers to ‘whether customers receive a fair economic or social outcome after complaining about a service problem’ ( Liao, 2007). Procedural justice refers to ‘the justice meted out regarding the policies and procedures used to resolve the complaints.’ Informational justice refers to ‘the adequacy of information and communication provided.’ Interpersonal justice is the sensitivity and respect employees display in handling complaints.

*Job demands*

Focal construct. Job demands are defined as ‘those physical, social, or organizational aspects of the job that require sustained physical or mental effort and are therefore associated with certain psychological costs and include aspects such as workload, time pressure, and difficult work environments’ ( Crawford *et al.*, 2010; p. 835).

*Job resources*

Focal Construct. Job resources are defined as ‘those aspects of the job that are functional in achieving work goals, stimulating personal growth and development, and reducing job demands, and they are associated with physiological and psychological costs and include aspects such as job control, opportunities for development, participation in decision making, task variety, feedback, and social support’ ( Crawford *et al.*, 2010; p. 838–836).

In the context of employee recovery performance, the relevant job resources are training, rewards, supportive management, and service technology support ( Rod & Ashill, 2009). These job resources are relevant in service management ( Babakus *et al.*, 2003; Singh, 2000). Job demands are conceptualized as either role stressors ( Ashill & Rod, 2011) or social stressors ( Dormann & Zapf, 2004). If the construct of job demands is conceptualized as a role stressor, its dimensions would be role conflict, role overload, and role conflict ( Rod & Ashill, 2009). If conceptualized as a social stressor, its dimensions would be customers’ intentions to harm employees, disproportionate customer expectations, ambiguous customer expectations, and hostility and undesirable behavior demonstrated by customers ( Choi *et al.*, 2014).

### Burnout

Burnout has been conceptualized ( Maslach & Jackson, 1981) as consisting of emotional exhaustion, depersonalization, and a reduced sense of personal accomplishment. When employees experience emotional exhaustion, they feel they have no more energy to live up to their employers’ demands or meet clients’ expectations. When employees experience a sense of depersonalization, they treat people as objects. Finally, when they experience a sense of inefficacy, they experience a reduced sense of personal accomplishment 

### Work engagement

Kahn (1992) conceptualized engagement as a function of individual characteristics, work context, and factors beyond the work context. Accordingly, he defined ‘personal engagement’ as ‘employing or expressing oneself physically, cognitively, and emotionally during work role performance.’ ( Kahn, 1992; Simpson, 2009). However, researchers later conceptualized engagement to include work-related factors alone ( Simpson, 2009). Accordingly, burnout and work engagement are conceptualized as two polar ends of a continuum ( Maslach *et al.*, 1997). Three dimensions were used to measure burnout (work engagement): exhaustion (energy), cynicism (involvement), and inefficiency (efficacy). Accordingly, this conceptualization of burnout and work engagement presupposes that employees are at any given point in this continuum at any given time. In other words, if an employee experiences emotional exhaustion (energy), he or she will also experience burnout (engagement). However, Schaufeli *et al.* (2002) differed in this conceptualization and argued that when an employee demonstrates low burnout, it cannot be understood as equivalent to high engagement and vice versa. They attributed this to the distinct nature of the two constructs. Therefore, they defined work engagement as a positive, fulfilling, work-related state of mind characterized by vigor, dedication, and absorption ( Schaufeli *et al.*, 2002). Accordingly, they conceptualized work engagement comprising three dimensions: vigor, dedication, and absorption Demerouti *et al.* (2001). While vigor and dedication capture emotional energy and involvement, the two dimensions conceptualized by Maslach *et al.* (1997), the dimension of absorption is a distinct dimension conceptualized by Demerouti *et al.* (2001).

### Problem statement

"What effect does the employee service recovery performance exercise have on customer justice perceptions if the employee service recovery performance is within the context of the JD-R Model and job demands and resources are nested within the context of the service recovery system?" This is a critical research problem addressed by this research.

**Review of literature**

The service recovery process aims to return customers to their state before service failure ( Mostafa *et al.*, 2015). Therefore, service recovery is the ‘organizational response to service failure’. Further, service failure is defined as ‘a service performance that falls below a customer’s expectations’ ( Hoffman & Bateson, 1997; Silber *et al.*, 2009). Though there are diverse conceptualizations of the construct of employee recovery performance, such as ‘the effectiveness of employees in satisfying complaining customers’ ( Vaerenbergh, 2016; p. 15) or ‘frontline employees’ perceptions of their abilities and actions to resolve service failures’, prior research has also identified six types of responses that organizations provide to execute service recovery ( Liao, 2007), viz., ‘tendering an apology, problem-solving, courteous behavior, providing an explanation, prompt handling, and offering extra compensation.’ However, caution is not misplaced in this context. Prior research has shown that customers are more concerned with outcome failures, such as the non-availability of service than process failures, that is, the absence of attention while providing service ( Smith *et al.*, 1999). Further, customer dissatisfaction will override service recovery strategies such as apologizing and providing compensation.

### Service recovery system, job resources and job demands relationship

Research on service recovery has attempted to address the issue of attaining the efficacy of the service recovery process and the resultant effect on customer-related variables by working on individual employees. In other words, the literature on service recovery has predominantly relied on individual analyses. Hospitality research on service recovery has predominantly applied a single level of analysis to understand the service management phenomenon ( Wong, 2016). However, as pointed out by Wong (2016), these studies “face a common research limitation in that they use data from a single level (mostly customers or employees) to infer social phenomena that exist at multiple levels.” Although parsimony is the specific advantage of single-level analysis, it cannot account for a multilevel understanding of a social phenomenon ( Aguinis *et al.*, 2011). In this connection, it is argued by scholars that the higher-level variables exert either direct or moderating effects on the lower-level variables ( Kozlowski & Klein, 2000). A given social phenomenon is embedded at multiple levels ( Wong, 2016). The theoretical underpinnings of this argument are provided by theories such as environment-fit theory and systems theory ( Wong, 2016), which argue that individuals are essentially social actors, and therefore, their behavior is nested within multiple levels of hierarchy in a social or organizational setting. Therefore, bridging a social phenomenon’s macro- and micro-perspectives gives rise to a critical understanding of this phenomenon. Therefore, this requires a multilevel design to address the issue of building relationships among constructs at different analysis levels. This avoids the probability of postulating the confounding effects of organization-level variables on the group- or individual-level variables or vice versa. Accordingly, this proposed research endeavor seeks to bridge this methodological gap in the research discourse on service recovery performance by conceptualizing and testing a multilevel model in which the effect of the macro-level construct of the service recovery system is tested on employee-level constructs of employee service recovery performance and its antecedents, that is, job resources and job demands, and the effect of the organization-level construct of the service recovery system and the individual-level construct of employee recovery performance on the customer-level construct of customer justice perceptions.

In this connection, the JD-R Model argues that the constructs of job resources and job demands can explain organizationally desirable employee-level outcomes through the mediating effects of employee burnout and work engagement. In this context, it is worth noting that job resources and demands are nested within the unit- or organization-level constructs. In this regard, the multilevel relationship, which involves a top-down process between the service recovery system and job resources and job demands, has been mooted in prior research. However, the hospitality sector has not empirically tested and validated the proposed relationship between the service recovery system, job resources, and job demands.

### Employee service recovery performance: customer justice perceptions relationship

Service recovery strategies operationalized in the construct of employee service recovery performance are essential because customers view them in the cost-benefit equation resulting from registering a complaint. As cost-benefit analysis is the basic premise of justice theory ( Del Rio-Lanza *et al.*, 2009), employees’ actions to mitigate service failures will be subjected to a process of evaluation of the justice meted out. Scholars have argued that customer justice perceptions determine service recovery ( Liao, 2007).

### Theoretical premises: a multiple-needs perspective of justice

The importance of customer justice perceptions can be understood from the multiple needs perspective of justice ( Cropanzano *et al.*, 2001), which argues that justice perceptions should be situated against the satisfaction of three human needs: instrumental, relational, and moral. While instrumental needs pertain to controlling the environment and accessing long-term benefits, relational needs refer to human needs for self-regard and social status, and moral virtue needs are those human needs that lead to aspiration for ‘human dignity and a virtuous life’ ( Cropanzano *et al.*, 2001; p. 175). Any experience of injustice will threaten these needs, and the individuals will adopt ‘defensive behaviors’ ( Liao, 2007; p. 476). Therefore, the efficacy of employee recovery performance consists of ensuring the satisfaction of these human needs so that the negative affect and resultant defensive behaviors do not manifest in customers.

It is essential to note those mentioned above, i.e., distributive justice, procedural justice, informational justice, and interactional justice ( Liao, 2007). Distributive justice is about providing the ‘just share’ ( Cropanzano, 2001; p. 37) regarding the output-input ratio of an employee’s performance-reward relationship. Equity theory explains the processes and consequences of inequity in an effort-reward relationship. It is interesting to note the disproportionate rewards concerning efforts that result in dissatisfaction and the disproportionate efforts and performance caused by over-rewarded efforts. This is interesting in the context of employee recovery performance, as it implies that customers are highly dissatisfied if they experience inequity in their treatment by employees.

Further, employee recovery performance is related to customer perceptions of procedural justice ( Thibaut & Walker, 1975). Customers are likely to be offended if they feel that policies and procedures are not correctly followed, even as they register their complaints in the event of a service failure. Furthermore, customers would get offended if they did not provide sufficient information and were not kept in the communication loop, even if their complaints were handled. Furthermore, employee recovery performance is also linked with interactional or interpersonal justice perceptions that emanate from the treatment meted out by employees to customers in the context of employee recovery performance ( Greenberg, 1993).

### Theoretical premises: cognition-affect-attitude theory

In this connection, the cognition-affect-attitude theory also provides a theoretical explanation for conceptualizing the relationship between employee recovery performance and customer justice perceptions. To substantiate this argument, scholars have argued that while the idea of perceived justice is the result of the cognitive evaluation ( Schoefer & Diamantopoulos, 2008) of employee recovery performance, satisfaction with service recovery is interpreted as the ‘affective response’ ( Liao, 2007) of customers towards employee recovery performance ( Davidow, 2000), and the positive attitude of customers that they develop towards the organization, for example, towards its service recovery system, resulting from employee recovery performance is considered to be the attitudinal aspect of customer response. Therefore, this theory provides conceptual underpinnings that explain the positive association between employee recovery performance and customer justice perceptions.

It is important to note that these types of customer justice perceptions are not isolated in nature. All of them are the dimensions of a single construct, customer justice perceptions, whose essential underlying theme is a sense of fairness ( Colquitt *et al.*, 2013). Therefore, employee recovery performance will be successful in ensuring the emergence of a sense of fairness among customers if they feel that their complaints and grievances are appropriately addressed in terms of correction of service failure and the reversal of service failure; fair procedures are followed in addressing the complaints; policies and procedures are put in place and duly communicated; and employees demonstrate fairness and dignity in their relationship with them.

### The need to study the employee service recovery performance-customer justice perceptions relationship.

Scholarly research on customer justice perceptions investigated the effect of this construct on several other outcome variables when those outcome variables were used as mediators in their respective models. Those variables which were posited to be the outcomes of customer justice perceptions, though as mediators, are the constructs such as service evaluation ( Aurier & Siadou-Martin, 2007), emotions ( Kim & Tang, 2016), negative emotions ( del Río-Lanza, Vázquez-Casielles & Díaz-Martín, 2009; Nikbin & Hyun, 2014; Kim & Tang, 2016), positive emotions ( Chebat & Slusarczyk, 2005; DeWitt, Nguyen & Marshall, 2008; Kim & Tang, 2016), satisfaction ( MartÃ­nez-Tur *et al.*, 2001; Teo Thompson & Lim Vivien, 2001; Maxham & Netemeyer, 2002; Davidow, 2003; Witrz & Mattila, 2004; Kim, Kim & Kim, 2009; Vázquez-Casielles *et al.*, 2010; de Matos, Vieira & Veiga, 2012; Xu *et al.*, 2014; Abbas, Abdullateef & Mokhtar, 2015; Zhou, 2015), credibility ( Vázquez-Casielles *et al.*, 2010), benevolence ( Vázquez-Casielles *et al.*, 2010), attitude towards complaining ( Ro & Olson, 2014), service failure attributes ( Witrz & Mattila, 2004), word of mouth valence ( Davidow, 2003), word of mouth dissemination ( Davidow, 2003), trust ( Kim & Kandampully, no date), commitment ( Kim & Kandampully, no date), customer affection ( Choi & Choi, 2013), customer loyalty ( Choi & Choi, 2013), privacy concern ( Zhou, 2015), flow ( Zhou, 2015), overall satisfaction ( Maxham & Netemeyer, 2002), satisfaction with recovery ( Liao, 2007; Mostafa *et al.*, 2015), and recovery disconfirmation ( McCollough, Berry & Yadav, 2000). All of these outcome variables highlight the importance of studying customer justice perceptions. Therefore, this Study posits customer justice perceptions as the outcome variable of the model that it intends to investigate.

Prior research on customer justice perceptions has investigated the effect of constructs such as company characteristics ( Homburg, Fürst & Koschate, 2009), over-reward ( Söderlund & Colliander, 2015), type of failure ( Masnita Siagian & Triyowati, 2015), locus of attribution ( Masnita Siagian & Triyowati, 2015), and customer characteristics ( Homburg, Fürst & Koschate, 2009) in their role as antecedents of customer justice perceptions. Studies that have examined the role of employee service recovery performance are scarce, except for the Study by Siagian and Triyowati (2015), who conceptualized employee service recovery performance as ‘recovery strategies.’ Therefore, this research seeks to bridge this gap by positing customer justice perceptions as the outcome variable of employee service recovery performance in the hospitality sector.

An examination of prior research on employee service recovery performance reveals that prior studies have investigated its effects on outcome variables, such as satisfaction ( McCollough, Berry, & Yadav, 2000; Wong, 2004; Roschk & Kaiser, 2013); service assessment ( Wong, 2004); repurchase intention ( Liao, 2007); corporate image ( Mostafa *et al.*, 2015); and continuity ( Selnes, 1998). However, studies on the effects of employee recovery performance on customer justice perceptions need to be conducted. This Study seeks to bridge this research gap.

### Antecedents of employee service recovery performance

Research discourse on employee recovery performance has examined the impact of its antecedents, such as service failure severity ( Weun *et al.*, 2006), formality ( Smith, Karwan, & Markland, 2009), knowledge sourcing behavior ( van der Heijden *et al.*, 2013), disproportionate customer expectations ( Choi *et al.*, 2014), job demand ( Rod & Ashill, 2009), ideas for improvement ( van der Heijden *et al.*, 2013), and emotional exhaustion ( Rod & Ashill, 2009; Choi *et al.*, 2014). Service failure severity, knowledge-sourcing behavior, and ideas for improvement pertain to frontline employees’ cognitive and behavioral aspects. However, disproportionate customer expectations and job demand belong to those factors that are external to frontline employees. Such categorization of antecedent constructs will open up a holistic understanding of the antecedents of employee recovery performance. Therefore, there is a gap in research on the possible effects of job resources and demands as antecedents of employee service recovery performance.

### Theoretical underpinnings: the relationship among job resources, job demands, burnout, and work engagement

The DCM, ERI, and JD-R models theorize the relationships among job resources, job demands, burnout, and work engagement. The JD-R Model is essentially a model of employee well-being. Researchers have proposed this model to overcome the limitations of two earlier models of employee well-being: the DCM ( Karasek, 1998) and the ERI model ( Siegrist, 1996).

### DCM

The DCM argued that employees would experience stress and a reduced sense of well-being if they felt that their control over their jobs was less than the demands of their jobs. Although this model has been predominantly applied to explain job stress, the related stream of research in this area has two exciting implications ( Bakker & Demerouti, 2007). First, strong support for the strain hypothesis results from an imbalance between job demands and control. Second, there is inconsistency regarding the buffering hypothesis regarding the moderating effect of job control exercises on the relationship between job demands and stress.

### ERI model

The ERI Model sought to explain the phenomenon of employee well-being from the perspective of an effort-reward imbalance. If high job efforts result from excessive job demands and the rewards are relatively low, it would result in a higher degree of ‘arousal,’ a physiological phenomenon, and the resulting stress. This is consistent with the equity theory’s low outcome/high input type of inequity. However, this model argues that the personal commitment of employees to their work and the organization, which results from employees’ desire to be valued by ‘significant others and the sense of esteem that they experience in this process, is expected to moderate the relationship between perceived inequity that results from effort-reward imbalance and stress.

### Evaluation of DCM, ERI, and JD-R models

The strengths and weaknesses of the DCM and ERI models are characterized. For example, the strength of both models is their simplicity, as they explain job stress as a function of only a few constructs. As has already been discussed, while the DCM Model employs two constructs (i.e., job demands and job control), the ERI Model uses two constructs (i.e., effort and reward) to explain the emergence of job stress and, consequently, the reduction in employee well-being. However, scholars argue that the simplicity of models constitutes their weakness ( Bakker & Demerouti, 2007). This is because it is not adequate to account for only two factors to explain the phenomenon of job stress: the consequent burnout and the engagement level. Therefore, researchers have listed several factors that are part of job resources and demands. Furthermore, these models do not incorporate factors such as autonomy and task characteristics.

### JD-R model

The JD-R model incorporates more aspects of job resources and demands than the DCM and ERI models. Accordingly, the construct of job resources is defined as “those physical, psychological, social, or organizational aspects of the job that are either/or functional in achieving work goals; reduce job demands and the associated physiological and psychological costs; and thus stimulate personal growth, learning, and development” ( Bakker & Demerouti, 2007; p. 312). This conceptualization of job resources is consistent with the job characteristics model ( Hackman & Oldham, 1980) and conservation of resources theory ( Hobfoll, 2001). The conceptualization of job resources aligns with the job characteristics model because it incorporates the conceptualization of job resources in terms of task characteristics, such as skill variety, task identity, task significance, autonomy, and performance feedback. Further, the conceptualization of job resources is consistent with the conservation of resources theory, as different types of job resources are viewed as fundamental to maintaining resources and achieving new and valued resources ( Bakker & Demerouti, 2007; p. 312).

### Job demands: employee service recovery performance relationship

The effect of job demands on employee recovery performance ( Rod & Ashill, 2009; Ashill & Rod, 2011) has been empirically demonstrated, in addition to its effects on other constructs such as turnover intention ( Karatepe & Sokmen, 2006; Ashill & Rod, 2011), job satisfaction ( Karatepe & Sokmen, 2006; Rod, Ashill, and Carruthers, 2008), organizational commitment ( Rod *et al.*, 2008), organizational outcomes ( Bakker & Demerouti, 2007), and job performance ( Babakus, Yavas & Ashill, 2009). However, the effect of job demands on employee recovery performance is not necessarily construed as direct in all the studies. Many studies have demonstrated the mediating effect of job demands on employee recovery performance, as demonstrated by studies that have shown mediating effects of constructs such as emotional exhaustion ( Babakus, Yavas, & Ashill, 2009; Rod & Ashill, 2009; Ashill & Rod, 2011), depersonalization ( Ashill & Rod, 2011), job satisfaction ( Ashill & Rod, 2011), leadership ( Schaufeli, 2015), employee service recovery performance ( Karatepe, 2006; Karatepe & Sokmen, 2006; Rod, Ashill & Carruthers, 2008), and strain ( Bakker & Demerouti, 2007). The implication of studies investigating the mediating effects of job demands on employee recovery performance is that job demands, an external variable, have a detrimental effect on the ‘internal’ variables, which would impact employee recovery performance.

### Job resources: employee service recovery performance relationship

Prior research has shown that the construct of job resources moderates the negative effects of job demands on ‘internal’ emotional and cognitive states. Further, the construct of job resources is proposed to create a positive ‘emotional state.’ Therefore, the construct of job resources is conceptualized as the exact opposite of job demands in terms of its impact on frontline employees. In this regard, prior research has investigated the effect of job resources on several dependent variables such as FLE service recovery ( Babakus *et al.*, 2003; Rod & Ashill, 2009), employee outcomes ( Schaufeli, 2015), peer contacts ( Hakanen, Bakker & Demerouti, 2006), engagement ( Crawford, Lepine & Rich, 2010), organizational outcomes ( Crawford, Lepine & Rich, 2010), turnover intention ( Babakus, Yavas & Ashill, 2009), and organizational performance ( Bakker & Demerouti, 2008). Many studies have conceptualized the impact of job resources on the respective dependent variables by conceptualizing the mediating effects of depersonalization ( Rod & Ashill, 2009), burnout ( Babakus, Yavas & Ashill, 2009; Schaufeli, 2015), affective commitment ( Babakus *et al.*, 2003), job satisfaction ( Babakus *et al.*, 2003), motivation ( Bakker & Demerouti, 2007), and work engagement ( Bakker & Demerouti, 2008). We carefully observed these mediating variables and realized that all of these mediating constructs were emotional or cognitive states. In other words, they are all ‘internal’ variables. Therefore, job resources and demands are conceptualized to lead to emotional states such as work engagement and burnout.

Against the backdrop of the above-discussed relationships between job demands and employee recovery performance and between job resources and employee recovery performance, the following research issues emerge:

The direct effect of job resources on employee service recovery performance

Direct effect of job demands on employee service recovery performance

Interactive effects of low job resources and high job demands on employee service recovery performance

Interactive effects of high job resources and low job demands on employee service recovery performance

Effects on employee service recovery performance if both job resources and job demands are either high or low

**Figure 2.**
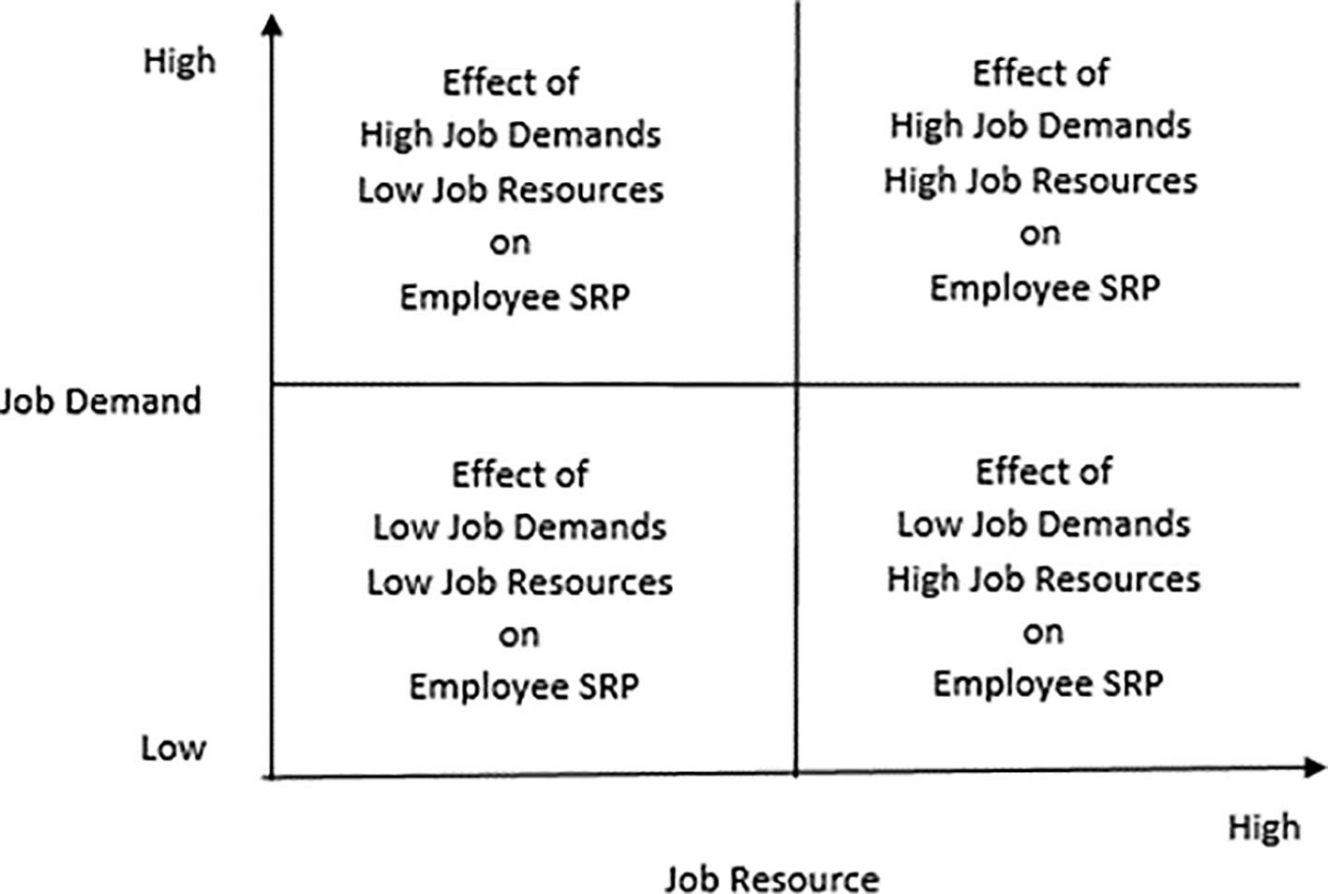
The four quadrants that capture the interactive effects of job resources and job demands on employee service recovery performance.

The above issues can be represented in a 2 × 2 matrix that captures the abovementioned research ( Figure 2).

### Job demands and job resources: burnout and work engagement relationships

The JD-R model argues that two processes set themselves in motion as consequences of job resources and demands ( Schaufeli, Bakker, & Rhenen, 2009). These are the strain and motivation processes ( Bakker & Demerouti, 2007). The compensatory regulation-control model ( Hockey, 1997) argues that increased job demands lead to a corresponding increase in employee effort to meet these demands, which would entail physical and psychological costs, resulting in burnout. Contrary to the straining process, self-determination theory argues that job resources contribute to fulfilling the human needs of autonomy, relatedness, and competence ( Ryan & Deci, 2000), which would thus initiate the motivational process. The rationale for the motivational potential of job resources is explained by the effort-recovery approach, which argues that employees increase their efforts if they perceive that there are enough job resources to enable them to succeed in reaching their work-related goals ( Meijman & Mulder, 1998).

### Job resources: work engagement relationship

Research on the consequences of job resources and job demands ( Bakker & Demerouti, 2007) has argued that these two constructs positively impact employee performance through the mediating processes of burnout and work engagement. This phenomenon is operationalized by theories such as the conservation of resources theory ( Hobfoll, 2011). This theory is widely used to explain the phenomenon of work engagement among employees ( Stock, Jong, & Zacharias, 2017). This theory is woven around the acquisition, maintenance, and fostering of resources that are defined as “those objects, personal characteristics, conditions, or energies that an individual values or that serve as a means for the attainment of these objects, personal characteristics, conditions, or energies” ( Hobfoll, 2011; p. 516). The arguments of the theory, as applicable to the job resources-work engagement relationship, can be articulated as follows: First, the attempt to prevent the probable loss of job resources would create “emotional labor” and thus lead to the creation of emotional energy among employees.

Consequently, the role of employees’ self-identification with work ( Chen *et al.*, 2013) comes into being. In other words, the emotional energy of employees created through their ‘emotional labor’ leads to role identity and consequent work engagement among employees ( Rich *et al.*, 2010). This proposition is also articulated in the frontline employees’ context ( Estrada, Isen, & Young, 1994).

### Job demands and burnout relationships

Prior research has proved that an antecedent-consequent relationship exists between job demands and emotional exhaustion. Among these two constructs, job demands are ‘external’ to frontline employees, whereas emotional exhaustion is ‘internal.’ Research on job demands and their effects has argued that they directly affect employee burnout. The ‘challenge stressors’ increase burnout among frontline employees with high achievement orientation. Further, ‘hindrance stressors’ increase emotional exhaustion just as ‘challenge stressors’ increase emotional exhaustion. Therefore, a higher intensity of all dimensions of job demands (i.e., workload, physical demands, emotional demands, and work-home interference) is expected to increase burnout among frontline employees.

### Theoretical underpinnings: interactive effects of job resources and job demands on burnout and work engagement

The JD-R Model states that one can situate work engagement and burnout within the framework of two general categories: job resources and job demands, regardless of differences in organizational or occupational characteristics ( Crawford, LePine, & Rich, 2010). The JD-R Model has overwhelmingly influenced the literature on work engagement and burnout, as this theoretical perspective is the dominant narrative in research discourse on work engagement and burnout ( Bakker & Demerouti, 2007). In this context, the JD-R Model states that job resources influence the extent of work engagement, and job demands influence the degree of burnout.

The research discourse on the much-hypothesized relationships between job demands and burnout and between job resources and work engagement is not as straightforward as it seems. In this regard, prior research ( Crawford *et al.*, 2010) has shown that job demands should be differentiated regarding challenge and hindrance stressors, even though it is well known that job demands initiate stress responses among employees that would eventually lead to burnout. Although both stressors lead to burnout, the differentiated job demands perspective argues that challenge stressors initiate positive emotions such as excitement, exhilaration, and eagerness, which lead to greater work engagement, as the source of challenge stressors would initiate perceptions of growth and learning. This is also consistent with the job characteristics model, which argues that challenging work leads to a sense of meaningfulness and personal responsibility ( Hackman & Oldham, 1980). Therefore, employees with higher organizational responsibilities are likely to feel the presence of challenges in their work and perceive a higher sense of personal accomplishment ( Kahn, 1992; Macey & Schneider, 2008). As a result, this category of job demands is likely to influence work engagement, although this does not rule out the possibility of burnout as the probability of expending one’s emotional energy in these high-performing work roles still exists. Therefore, the possibility of emotional exhaustion cannot be ruled out ( Crawford *et al.*, 2010).

Further, types of job demands that can be categorized as hindrance stressors initiate negative emotions such as fear, anxiety, and anger. Consequently, employees enter a spiral of emotional coping styles. Accordingly, they engage in defensive behaviors such as rationalization and withdrawal. The differentiated job demands perspective does not advocate a simplistic assertion that increased job demands will lead to increased burnout. Therefore, it advocates distinguishing between challenge and hindrance demands while conceptualizing job demands. This distinction within the broader category of job demands will lead to the proposition that challenging demands will increase work engagement, even as they increase burnout, as they can potentially increase emotional exhaustion while employees attempt to meet demands. The challenge demands lead to positive emotions that initiate a problem-focused coping style, leading to higher work engagement.

**Figure 3.**
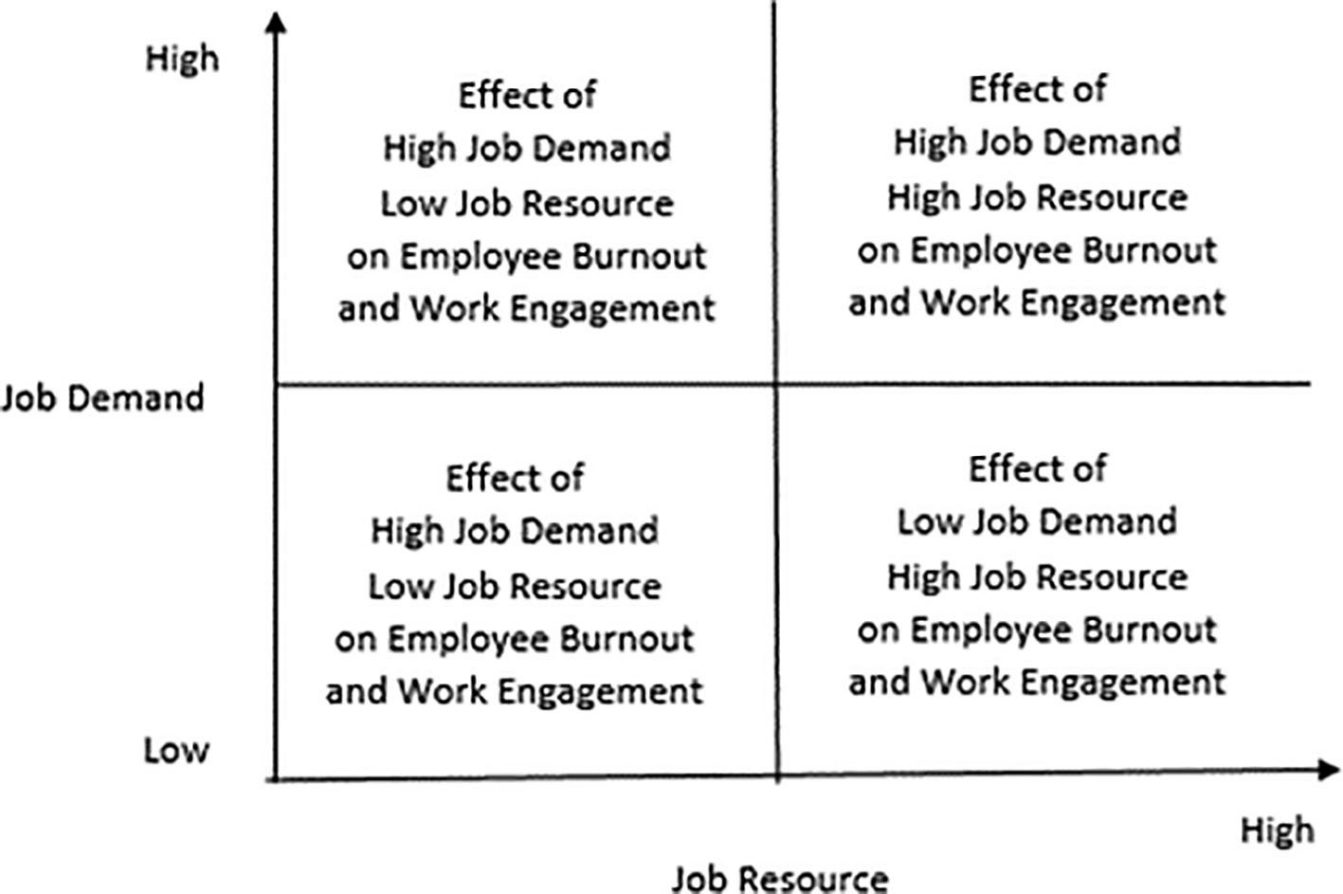
The four quadrants that capture the interactive effects of job resources and job demands on burnout and work engagement.

In contrast, hindrance stressors lead to negative emotions that initiate an emotion-coping process, reducing work engagement and increasing burnout. The differentiated job demands perspective of Crawford *et al.* (2010) proposes that job resources perform two functions. First, they initiate motivational processes that inspire employees to increase work engagement. Second, they also reduce the strain that employees may experience resulting from potential resource depletion over time. The issues discussed above can be seen in Figure 3.

### Interactive effects of job resources and job demands on employee burnout and work engagement

Although the interactive effect of job resources and job demands on employee recovery performance in the hospitality sector has not been studied, their interactive effect on burnout and work engagement has been studied in other organizational settings. In this context, prior research has also proposed a differentiated perspective on job demands by distinguishing between challenge demands and stressors and hindrance stressors within the broader construct category of job demands. According to this perspective, job demands do not necessarily lead to burnout only, as ordinarily proposed; they can also lead to increased work engagement to the extent of the presence of challenge demands within the broader category of the construct ‘job demands.’

This study seeks to bridge this research gap. Therefore, this Study examines the effects of job demands on burnout and work engagement and the effects of job resources on burnout and engagement when the interactive effects of job demands and job resources are considered. It is essential to study the effect on employee recovery performance because frontline employees’ service recovery behaviors are likely affected by their emotional states, such as burnout and work engagement. Therefore, the following research issues have emerged:

### Burnout: employee service recovery performance relationship

The importance of burnout can be understood from the fact that its impact is investigated on a wide range of variables such as trust ( Ledgerwood, Crotts, & Everett, 1998), cohesion ( Ledgerwood, Crotts, & Everett, 1998), reward ( Ledgerwood, Crotts, & Everett, 1998), willingness to deliver quality service ( Law & Tam, 2007), increased job demand ( Schaufeli *et al.*, 2009), job performance ( Maslach, Schaufeli & Leiter, 2001), health ( Maslach, Schaufeli & Leiter, 2001), and personal outcomes ( Maslach & Jackson, 1981). Although the direct effect of burnout on employee service recovery performance ( Karatepe, 2006) has been studied, its effect as a mediator and work engagement on employee recovery performance when job resources and job demands interact has not been studied. However, the hospitality sector has not investigated the effect of burnout on frontline employees’ service recovery performance.

### Work engagement: employee service recovery performance relationship

Engagement is motivational because it simultaneously invests individuals’ physical efforts, cognitive applications, and emotional investments in work roles ( Kahn, 1992; Rich *et al.*, 2010). Therefore, engagement brings deeper aspects of the human self into the workplace ( Rich *et al.*, 2010). There are three reasons for conceptualizing the positive relationship between work engagement and employee recovery performance. First, work engagement improves employee recovery by enabling frontline employees to expend their physical efforts for an extended period. Second, it enables the cognitive application of frontline employees’ attention to recovery performance in a vigilant and focused manner. Third, work engagement among frontline employees leads to the emotional investment of their energies to connect with their coworkers and to meet the emotional demands of their work roles so that there will be authentic performance’ ( Rich *et al.*, 2010).

Similarly, even though the effect of work engagement on increased job resources ( Schaufeli *et al.*, 2009) and organizational commitment ( Jung & Yoon, 2016) has already been investigated in the literature, its direct effect on frontline employees’ recovery performance in the hospitality sector has not been investigated. Although prior research has examined the effect of several constructs in their role as antecedents of employee service recovery performance, no study in the hospitality sector has investigated the direct effect of frontline employees’ work engagement on their service recovery performance.

### Interactive effects of burnout and work engagement on employee service recovery performance

Accordingly, the possible interactive effects among burnout, work engagement, and employee service recovery performance would throw up the following research issues:

Interactive effects of low burnout and high engagement on employee service recovery performance

Interactive effects of low work engagement and high burnout on employee service recovery performance

Interactive effects of high burnout and high work engagement on employee service recovery performance

Interactive effects of low burnout and low work engagement on employee service recovery performance

**Figure 4.**
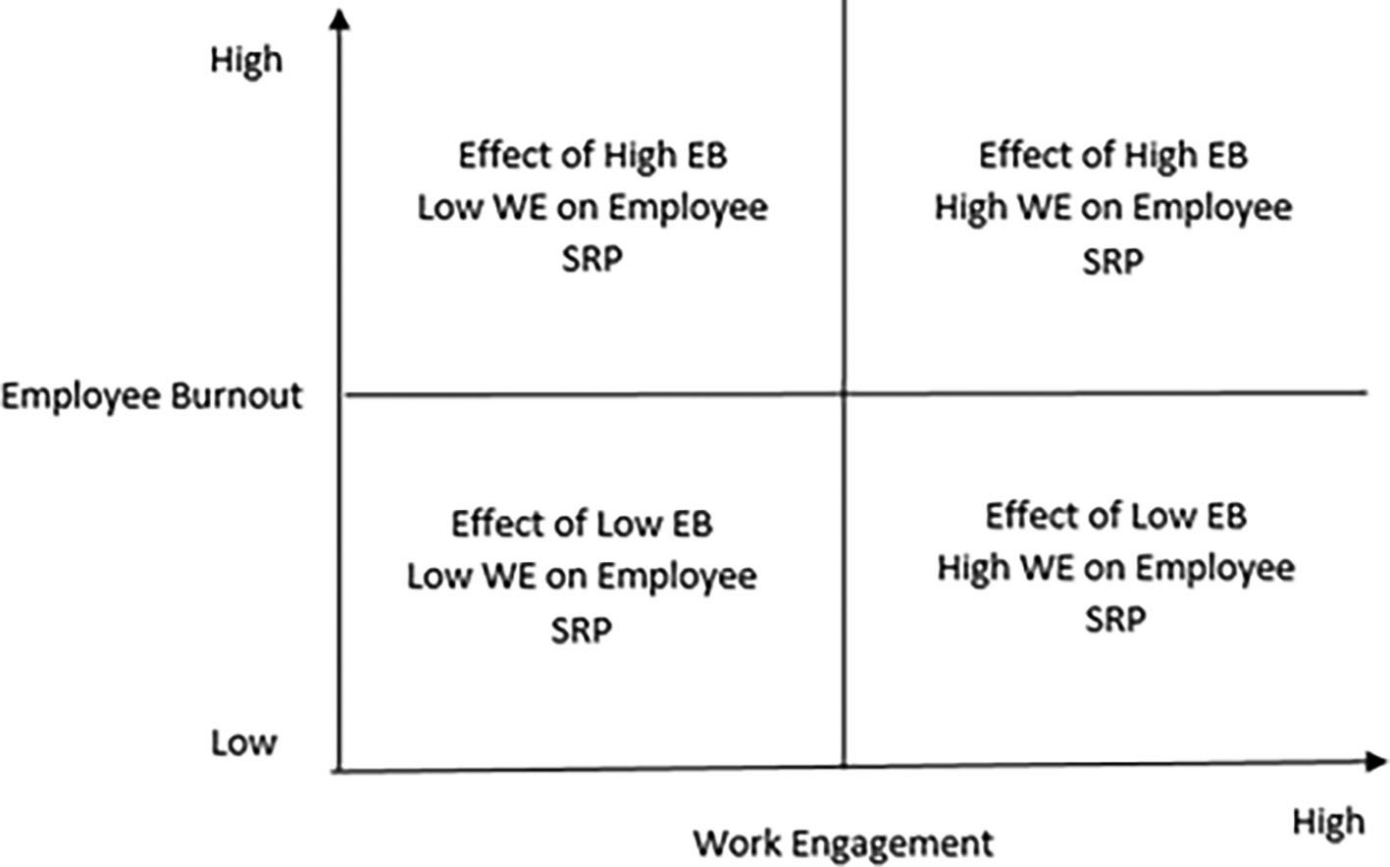
The four quadrants that represent the interactive effects of burnout and work engagement on employee service recovery performance.

The research mentioned above is captured in Figure 4.

The service recovery process aims to return customers to their state before service failure ( Mostafa *et al*., 2015). This research endeavour seeks to bridge this methodological gap in the research discourse on service recovery performance by conceptualising and testing a multilevel model in which the effect of the macro-level construct of the service recovery system is tested on employee-level constructs of employee service recovery performance and its antecedents, that is, job resources and job demands, and the effect of the organisation-level construct of the service recovery system and the individual-level construct of employee recovery performance on the customer-level construct of customer justice perceptions.

The JD-R model argues that the constructs of job resources and job demands can explain organizationally desirable employee-level outcomes through the mediating effects of employee burnout and work engagement. The hospitality sector has not empirically tested and validated the proposed relationship between the service recovery system, job resources, and job demands.

Finally, this paper focuses on three main issues in service recovery: 1) the importance of a well-understood approach to service recovery; 2) the need for a strong theoretical foundation; and 3) the role that service recovery plays in society. This paper presents a united view of service recovery management across fields and levels of theoretical and analytical analysis.

### Potential contributions of the research

The JD-R model has been widely used to understand the relationship between job demands and resources and employee well-being and performance. This research takes a step further by examining the impact of JD-R model constructs on employee service recovery performance. The findings suggest that job demands such as emotional dissonance and workload negatively affect service recovery performance, while job resources such as social support and autonomy positively impact it. Moreover, this study highlights the importance of considering multi-actor designs in construct measurement to enhance the accuracy of results. By doing so, this research contributes to a better understanding of the antecedents and consequences of service recovery performance within the context of a service recovery system at the unit level. Overall, these findings have implications for organisations seeking to improve their service quality by enhancing their employees’ service recovery performance through effective job design and resource allocation.

### Future research opportunities

The following research gaps are evident from the discussion above: First, there is a need to determine the relationship between the constructs of the service recovery system and job resources and demands in the hospitality sector, which has remained unexplored. Second, the relationship between employee service recovery performance and customer justice perceptions in the hospitality sector remains unexplored. Third, the direct and interactive effects of job resources and demands on employee recovery performance among frontline employees in the hospitality sector remain to be examined. Fourth, the direct and interactive effects of job resources and job demands on employee burnout and work engagement among frontline employees in the hospitality sector remain unexplored. Fifth, the direct and interactive effects of burnout and work engagement on employee service recovery performance among frontline employees in the hospitality sector remain to be investigated. Finally, multilevel modeling of the unit-level construct of the service recovery system and its effect on the customer-level construct of customer justice perceptions has not been attempted while empirically testing the antecedent-consequent relationship among job resources and job demands, employee burnout and work engagement, and employee service recovery performance still needs to be built. For example, a study could be conducted to investigate how training programs and organizational support can enhance service recovery performance among hotel receptionists who often interact with disgruntled guests. By collecting data on job resources, job demands, burnout, work engagement, and service recovery system at the unit level, researchers could examine how these factors impact frontline employees’ ability to restore guest satisfaction and subsequently affect customers’ perceptions of justice. Such insights could inform the development of evidence-based interventions that enhance the quality of service recovery

## Conclusions

This study presents a cohesive perspective on service recovery management across disciplines, levels of theory, and degrees of analysis. It organises this material into an interdisciplinary framework to help better understand the many disciplines looking at service recovery and offer suggestions on how organisations may build up service recovery procedures. The future research questions presented in this article can improve the research efficacy of academics interested in service recovery, and this synthesis and integrative framework will help researchers and managers think differently about service recovery and how it can be approached in a more comprehensive and effective manner. Overall, this article provides valuable insights and recommendations for organisations looking to improve their service recovery processes and for researchers seeking to advance the field of service recovery.

## Data Availability

No data are associated with this article.
