# Supplementary material for: Linkages between service recovery system and customer justice perceptions: A multi-level model of employee service recovery performance
Source: F1000Res. 2024 Oct 14;12:947. Originally published 2023 Aug 7. [Version 2] doi: 10.12688/f1000research.135103.2 (PMC11503003; doi:10.12688/f1000research.135103.2)
Supplement: Peer review history

# Reviewer report: Reviewer response for version 2

**DOI:** 10.5256/f1000research.172742.r331608

**Published:** 2024-10-24

**Authors:** Ngoni Courage Shereni

**Affiliations:** 1 School of Tourism and Hospitality Management, University of Johannesburg, Auckland Park, Gauteng, South Africa

I have gone through the revised manuscript and I am satisfied with the corrections made by the authors.

Is the review written in accessible language?

Yes

Are all factual statements correct and adequately supported by citations?

Partly

Are the conclusions drawn appropriate in the context of the current research literature?

Partly

Is the topic of the review discussed comprehensively in the context of the current literature?

Yes

Reviewer Expertise:

Sustainable Tourism, Marketing, human resource management, sharing economy

I confirm that I have read this submission and believe that I have an appropriate level of expertise to confirm that it is of an acceptable scientific standard.

# Reviewer report: Reviewer response for version 1

**DOI:** 10.5256/f1000research.148206.r195400

**Published:** 2024-09-26

**Authors:** Michael Sammanasu Joseph, Dr.Anjali Daisy

**Affiliations:** 1 Department of Management studies, St Joseph’s College Autonomous, Bharathidasan University, Tiruchirappalli, Tamil Nadu, India; 2 Department of management studies, St.Joseph's college Autonomous, Bharathidasan University, Tiruchirappalli, Tamil Nadu, India

In reviewing the manuscript titled “Linkages between Service Recovery System and  Customer Justice Perceptions: A Multi-Level Model of Employee Service Recovery    Performance”, it is evident that the authors have put forth a commendable effort in addressing the key aspects related to service recovery, customer satisfaction, and employee engagement.

 The article's strength lies in its comprehensive coverage of relevant literature from various fields, demonstrating a strong foundation of knowledge. The exploration of the relationships between service recovery, justice perceptions, employee engagement, and customer satisfaction is particularly noteworthy. However, there are several areas that require attention to enhance the quality and impact of the manuscript. The background and literature review sections, while extensive, lack focus on the specific themes and trends that connect to the proposed journal's theme.

 The  authors should consider refining these sections to clearly establish the relevance of their work within the broader context of the proposed journal. Additionally, the organization of the article can be improved to enhance its flow and coherence. The transitions between sections need to be smoother, ensuring that readers can follow the logical progression of ideas.

 This will help in maintaining the reader's engagement throughout the article. Moreover, while the authors have provided a comprehensive list of references, it's important for them to thoroughly integrate and discuss these references within the context of their arguments. This will demonstrate the critical evaluation of existing literature and establish the significance of their contributions. In terms of the strengths, the authors have effectively incorporated both theoretical and empirical research, contributing to the article's credibility. The use of real-world examples and cases adds practical relevance to the discussion. To address the aforementioned concerns, the authors are encouraged to revise the manuscript with a clear focus on refining the background, enhancing the organization, and ensuring the effective integration of references. These revisions will significantly enhance the manuscript's contribution to the proposed journal and its relevance to the target audience.

Is the review written in accessible language?

Yes

Are all factual statements correct and adequately supported by citations?

Yes

Are the conclusions drawn appropriate in the context of the current research literature?

Yes

Is the topic of the review discussed comprehensively in the context of the current literature?

Yes

Reviewer Expertise:

Human resource

We confirm that we have read this submission and believe that we have an appropriate level of expertise to confirm that it is of an acceptable scientific standard.

# Reviewer report: Reviewer response for version 1

**DOI:** 10.5256/f1000research.148206.r270144

**Published:** 2024-05-22

**Authors:** Ngoni Courage Shereni

**Affiliations:** 1 School of Tourism and Hospitality Management, University of Johannesburg, Auckland Park, Gauteng, South Africa

Abstract

 - I suggest that the Authors indicate the key findings of the study before highlighting the contribution of the study

 Introduction

 -The first line defines service recovery in direct quotes, can the authors provide the page number for the citation

 - The section 'Need for a standardized service recovery system'  and several other sections have no citations. There is a need to relate the arguments presented to existing literature

 Conceptual framework

 -While the framework clearly links service recovery with different variables there is also a need to ground these arguments in the literature. Readers would want to know what informs this framework.

 Conclusion

 This section should present the theoretical and practical implications of the research, limitations of the research and future research opportunities.

 Overall this is an interesting research that provides unique dimensions of service recovery. The researchers need to add current literature to substantiate their arguments

Is the review written in accessible language?

Yes

Are all factual statements correct and adequately supported by citations?

Partly

Are the conclusions drawn appropriate in the context of the current research literature?

Partly

Is the topic of the review discussed comprehensively in the context of the current literature?

Yes

Reviewer Expertise:

Sustainable Tourism, Marketing, human resource management, sharing economy

I confirm that I have read this submission and believe that I have an appropriate level of expertise to confirm that it is of an acceptable scientific standard, however I have significant reservations, as outlined above.

# Response

**Published:** 2024-06-23

**Authors:** Abhishek Rao

**Affiliations:** Commerce, Manipal Academy of Higher Education, Manipal, Karnataka, India

**Abstract**

**- I suggest that the Authors indicate the key findings of the study before highlighting the contribution of the study**

***Response:*** *We have addressed this issue in the below paragraph;*

 Past research aimed at service recovery has focused on actions that are taken to retain customers, and the possibility of overcoming the mistakes of service delivery,  though the multidimensional nature of the service recovery process has  begun to move beyond the complaint handling process. In this paper, we identify the most important issues addressed in service recovery and present a framework for addressing them

 We used an extensive integrative review methodology. Between 1988 and 2017 the number of articles in these journals was kept between 26 and 30. The majority took a business perspective to study services recovery, while a minority took an inter-disciplinarity perspective.

 The study's findings are expected to provide insights into the antecedents and consequences of service recovery performance, particularly how job resources and demands influence employee burnout and work engagement, which in turn affect service recovery performance and customer perceptions of justice. The research aims to contribute to the discourse on service recovery by building a multi-level model that explains the service recovery performance of frontline employees and the impact of the service recovery system on customer justice perceptions

 Thus, this research is expected to contribute to robust conclusions on the antecedents and consequences of employee service recovery performance nested within the unit-level construct, i.e., the service recovery system

**Introduction**

**The first line defines service recovery in direct quotes, can the authors provide the page number for the citation**

***Response:*** *Revised definition with page no;*

 Service recovery as “the actions taken by a service provider aimed at resolving failures” (Smith et al., 2009, p. 166).

**The section 'Need for a standardized service recovery system'  and several other sections have no citations. There is a need to relate the arguments presented to existing literature**

***Response:*** *Thank you for your feedback regarding the absence of citations in the section 'Need for a standardized service recovery system' and other sections of the document. We acknowledge the importance of grounding our arguments within the existing body of literature to provide a solid foundation for our research.*

 This overwhelming evidence that indicates the organizational importance of customer justice perceptions in the service recovery context has led to the increasing need to formalize the service recovery system that guides and trains frontline employees in their service recovery performance and places the systems and processes in this regard Smith, A. K., & Bolton, R. N. (2002). Formalizing and institutionalizing the service recovery system assumes importance in the context of its role in determining customer justice perceptions influenced by employee service recovery performance Mattila, A. S., & Patterson, P. G. (2004). Therefore, this research proposal intends to examine the impact of the service recovery system, conceptualized and operationalized at the organizational level, on the perceptions of customer justice that arise in the service recovery process

**Conceptual framework**

**-While the framework clearly links service recovery with different variables there is also a need to ground these arguments in the literature. Readers would want to know what informs this framework.**

***Response:*** *We appreciate your insightful feedback regarding the need to ground the framework within the existing literature. We understand the importance of providing a clear rationale for the framework's design, which is informed by a comprehensive review of the literature on service recovery, employee performance, and customer justice perceptions.*

 To address your concern, For instance, we have referenced studies that have established the link between service recovery systems and customer justice perceptions, as well as research that has explored the role of employee burnout and work engagement in service recovery performance.

 Moreover, we have included a section that discusses the theoretical underpinnings of the framework, citing specific theories and models that have informed our approach. This includes the Job Demands-Resource (JD-R) model, which is central to our understanding of how job resources and demands influence employee well-being and performance.

 We have also ensured that the framework's variables are clearly defined and that their interrelationships are explained with reference to empirical studies. This will help readers to understand the basis for the framework and how it contributes to the existing body of knowledge on service recovery.

 We are confident that these will provide a more robust foundation for our research proposal and will enhance the document's clarity and academic rigor. Thank you for your guidance, and we look forward to your continued feedback as we refine our work.

**Conclusion**

**This section should present the theoretical and practical implications of the research, limitations of the research and future research opportunities.**

***Response:*** *Thank you for your feedback regarding the section on conclusion. We understand the importance of providing a clear rationale. Here is the explanation given below*****

 Theoretical Implications:

 Our study contributes to the theoretical landscape by offering a novel perspective on service recovery that accounts for both organizational and individual-level factors. The proposed multi-level model bridges the gap between macro and micro perspectives, enhancing our understanding of the service recovery process. This integration of the JD-R model into service recovery research provides a foundation for future studies to build upon, potentially leading to the development of more nuanced theories in the field.

 Practical Implications:

 From a practical standpoint, the findings of this research can guide organizations in the hospitality sector to design more effective service recovery systems. By recognizing the importance of job resources and demands, managers can implement strategies to support frontline employees, thereby enhancing their ability to deliver satisfactory service recovery. This, in turn, can lead to improved customer satisfaction and loyalty, which are critical for the success of hospitality businesses.

 Limitations:

 It is important to acknowledge the limitations of this research. The study is based on an extensive integrative review, which may not capture the latest empirical findings. Additionally, the focus on the hospitality sector may limit the generalizability of the results to other industries. The research also relies on the JD-R model as a primary theoretical framework, which may not fully account for all the complexities of service recovery processes.

 Future Research Opportunities:

 This research opens up several avenues for future studies. Empirical testing of the proposed model is necessary to validate the relationships between service recovery systems, employee performance, and customer justice perceptions. Further research could also explore the role of additional organizational factors and individual differences in service recovery. Extending the research to other sectors could provide insights into the applicability of the model across different contexts.

 In conclusion, this research represents a significant step towards a deeper understanding of service recovery in the hospitality industry. By highlighting the importance of employee well-being and effective service recovery systems, we hope to inspire further research and practical applications that will benefit both employees and customers in the service industry.

**Overall this is an interesting research that provides unique dimensions of service recovery. The researchers need to add current literature to substantiate their arguments**

***Response:*** *We appreciate your positive feedback on our research and your suggestion to incorporate current literature to strengthen our arguments. We understand the importance of staying up-to-date with the latest findings in the field to ensure that our research is both relevant and robust.*

 To address your recommendation, we have conducted a thorough review of recent literature published within the last few years. We have identified several key studies that have advanced the understanding of service recovery, particularly in the context of the hospitality industry. These studies have explored various aspects of service recovery, including the impact of technology on recovery processes, the role of employee empowerment, and the influence of cultural factors on customer perceptions of justice.

 We have integrated these new findings into our document, ensuring that our framework and arguments are grounded in the most current research. This includes updating our literature review to reflect the latest developments and revising our theoretical framework to incorporate new insights from the recent literature.

 We believe that these additions will enhance the document's relevance and will provide a more comprehensive understanding of the service recovery landscape. We are committed to ensuring that our research remains at the forefront of academic discourse and practical application in the field of service recovery.

 Thank you for your guidance, and we look forward to your continued feedback as we finalize our work
